# The effect of increased lipoproteins levels on the disposition of vincristine in rat

**DOI:** 10.1186/s12944-016-0318-0

**Published:** 2016-09-09

**Authors:** Hadeel A. Khalil, Tarek S. Belal, Ahmed F. El-Yazbi, Dalia A. Hamdy

**Affiliations:** 1 Pharmaceutical Analytical Chemistry Department, Faculty of Pharmacy, Alexandria University, 1 El Khartoum Square, Alexandria, 21521 Egypt; 2 Department of Pharmacology & Toxicology, Faculty of Pharmacy, Alexandria University, Alexandria, 21521 Egypt; 3 Faculty of Medicine, the American University of Beirut, Lebanon, Alexandria, Egypt

**Keywords:** Hyperlipidemia, Acute lymphoblastic leukemia, Pharmacokinetics, Unbound fraction, Neurotoxicity

## Abstract

**Background:**

Vincristine (VCR), an antineoplastic agent, is a key component in the treatment of acute lymphoblastic leukemia, lymphomas, rhabdomyosarcoma, neuroblastoma, and Wilms’ tumor diseases. Recently, high incidence of hyperlipidemia was reported to be associated with allogenic hematopoietic stem cell transplantation and VCR/L-asparaginase therapy.

The aim of this study is to test the effects of incremental increase in lipoproteins levels on vincristine disposition in rat.

**Method:**

To study VCR pharmacokinetics and protein binding, rats (*n* = 25) were assigned to three groups, normal lipidemic (NL), intermediate (IHL) and extreme hyperlipidemic (HL). Hyperlipidemia was induced by ip injection of (1 g/Kg) poloxamer 407 in rats. Serial blood samples were collected using the pre-inserted jugular vein cannula for 72 h post VCR (0.15 mg/Kg) i.v. dose. VCR unbound fractions in NL, IHL and HL plasma were determined using ultrafiltration kits.

**Results:**

VCR demonstrated a rapid distribution phase (6–8 h) followed by a slower elimination phase with a mean elimination t½ of ~ 14 h. VCR exhibited moderate binding to plasma proteins ~ 83 %. It showed a relatively small Vc (~0.17 L/Kg) and a larger Vβ (1.53 L/Kg) indicating good tissue distribution. As the lipoproteins levels were increased, no significant changes were noted in VCR unbound fraction, plasma concentration, or volume of distribution indicating low affinity to lipoprotein binding. Induced HL also did not affect VCR elimination where similar VCR AUC_0-∞_, Cl and elimination phase t½ were reported along the different lipemic groups.

**Conclusion:**

Incremental increase in lipoprotein levels resulted in no significant effect on VCR disposition as such ALL malignant lymphoma and allogenic hematopoietic stem cell transplantation patients need not to worry about HL-VCR interaction. Whether, HL can potentiate another drug-drug or drug-disease interaction involving VCR warrants further studying and monitoring to ensure therapeutic safety and efficiency.

## Background

Acute lymphoblastic leukemia (ALL), characterized by immature lymphoid cells in the bone marrow, peripheral blood and other organs, has a high incidence in USA and worldwide [1]. It affects children and adults with median diagnosis age of 14 years [1]. ALL treatment show higher cure rates in children than adults and involves a complex and intensive therapy program. Said R et al. [1] vincristine (VCR) is an antineoplastic agent that acts by inhibiting microtubule formation in the miotic spindle causing cell death that may be accompanied by neurological side effect [2, 3]. It represents a key component in the ALL in children and adults and is currently the backbone of the induction, intensification, and consolidation phases in the treatment of this disease [4]. VCR is also used in combination with other oncolytic drugs for the treatment of Hodgkin’s disease, non–Hodgkin’s malignant lymphomas, rhabdomyosarcoma, neuroblastoma, and Wilms’ tumor [5]. VCR clinical efficacy is significantly dependent on its concentration and duration of exposure [1]. Unfortunately, it carries the potential for neurological toxicity that is both dose and duration dependent and is characterized by neuropathy, paresthesia, sensory deficits, muscle weakness, and rarely seizures [3, 6]. Hence, minimal alterations in VCR plasma concentrations might have serious consequences on its efficacy as well as its neurotoxicity.

After VCR intravenous injection, it is metabolized by liver CYP3A (mainly CYP3A4 and CYP3A5) and eliminated mainly in the bile with a low proportion excreted in the urine (about 8 to 15 %) [1, 3, 7] VCR reported clinical PK data showed a wide range of difference in the elimination phase t½ (range 155–5100 min), volume of distribution (V_β_) (range 57–420 L/m^2^) and clearance (range 82–482 mL/min/m^2^) among tested patients [1]. It has linear low affinity binding to plasma proteins, mostly AAG, with a reported unbound fraction of 0.5 [7]. Generally, VCR has a short distribution t½, large volume of distribution and long elimination phase t½ suggesting a high capacity to normal tissue binding which may result in limited tumor tissue exposure in vivo [1]. VCR also demonstrated high accumulation in most organs and tissues except the brain, eye and fat [1]. In rodents, VCR exhibited similar PK pattern where it was reported to have a biphasic plasma drug concentration decay patterns with a rapid distribution phase t½ ranging between 42–73 min followed by a slower elimination phase t½ of 14.3 ± 6.3 h. it showed a relatively large volume of distribution and a clearance of 0.12 ± 0.08 L/h/Kg [8].

Hyperlipidemia (HL) is a common pathological condition characterized by an abnormal elevation of plasma lipoproteins [9–11]. It is either caused by a defect in the genetic makeup or secondarily to other factors including diet, sedentary lifestyle habits, and certain drugs or diseases [12]. Worldwide increases in diabetes and obesity have increased the HL incidence in the last few years [9, 12]. A condition that poses an important clinical concern due to the sustained increases in low-density lipoprotein (LDL) that is a direct contributor to an increased risk of atherosclerosis, hypertension and ischemic heart disease [13, 14]. In addition, recent publications have clearly demonstrated the effect of HL on pharmacokinetics, pharmacodynamics and toxicodynamics of some drugs [11, 15–18]. This is owing to either reduced unbound fraction, altered metabolizing enzymes expression and/or enhanced lipoprotein receptor–mediated drug tissue uptake [11, 12, 16].

Recent reports have demonstrated a high incidence of HL in the first two years after allogenic hematopoietic stem cell transplantation [19]. In addition acute and severe hypertriglyceridemia (>10,000 mg/L of plasma triglyceride concentration) was shown to be associated with VCR/L-asparaginase treatment of ALL and malignant lymphoma patients [20]. This was suggested to be due to higher frequency of the apoE4/E3 phenotype in the patients with extreme hypertriglyceridemia as well as an increase in the apoCIII/apoCII ratio that results in inhibition of lipoprotein lipase enzyme leading to an accumulation of triglyceride-rich lipoproteins in plasma. Whether a potential interaction between VCR and HL exists is still unknown, however, a study of this interaction is crucial considering that HL has potential effect on metabolizing enzymes and transporters and that small alterations in VCR concentrations can affect its clinical efficacy and/or toxicity. As such, in the current manuscript we are testing the impact of different levels of plasma lipoproteins concentrations on the disposition of VCR regarding its plasma levels and protein binding in a polymer-induced HL rat model.

## Methods

### Materials and reagents

Vincristine powder (purity >99 %) was purchased from Selleckchem (Houston, TX, USA). Itraconazole was a kind gift from (Nifty Labs PVT LTD, Hyderabad, India). HPLC grade Methanol and acetonitrile (Fisher Scientific UK Limited, Loughborough, Leicestershire, UK), analytical grade potassium dihydrogen orthophosphate (Riedel-de-Haën, Germany) and high purity distilled water were used. Vinracine^®^ intravenous injection vials labelled to contain 1 mg/mL vincristine sulphate (EIMC United Pharmaceuticals, Cairo, Egypt), normal saline and heparin sodium for injection 5000 U/mL were purchased from the Egyptian market. Aerraine^®^ Isoflurane USP was also purchased from local market.

### Animals and pre-experimental procedures

Experimental protocols involving animals, were approved by the Ethics Committee, Faculty of Pharmacy, Alexandria University. Sprague–Dawley rats were used in the studies. All rats weighed between 150 and 250 g and were housed in temperature-controlled rooms with 12 h of light per day. The animals were fed a standard commercial rat chow containing 4.5 % fat (Tanta Oil and Soap JSC, Tanta, Egypt). Free access to food and water was permitted prior to experimentation. Rats were allocated into either normolipidemic (NL), intermediate hyperlipidemic (IHL) or extreme hyperlipidemic (HL) groups. HL was induced by single intraperitoneal injection of 1 g/kg of P407 (0.13 g/ml solution in normal saline). To ensure the proper injection of P407, the animals were lightly anaesthetized using isoflurane, and then allowed to recover. The rats were prepared for i.v dosing by jugular vein cannulation. For each rat, a single jugular vein was catheterized with Silastic Laboratory Tubing (Dow Corning Corporation, Midland, MI) under isoflurane/O_2_ anesthesia, administered by anesthetic machine. The cannula was filled with locking solution containing 100U/ml heparin in 0.9 % saline. After implantation, the rats were transferred to their regular holding cages and allowed free access to water, but food was withheld overnight so that drug would be administered in the fasted state. The next morning, normolipidemic rats were transferred to the metabolic cages and dosed with i.v. VCR. The hyperlipidemic and intermediate hyperlipidemic groups were dosed VCR after 36 h and 72 h from the P407 injection, respectively.

### Drug administration and sample collection

The intravenous solution was prepared by diluting Vinracine^®^ (1 mg/mL) intravenous injection vials with normal saline to reach a final concentration of 0.1 mg/mL. Rat groups (*n* = 5–7 each) received 0.15 mg/kg VCR i.v. immediately followed by injection of sterile normal saline. At the time of first sample withdrawal, the first 0.2 mL volume of blood was discarded. Food was provided to animals 2 h after the dose administration.

Serial blood samples (0.15–0.25 mL) were collected using the jugular vein cannula at 0.083, 0.167, 0.333, 0.5, 0.75, 1, 2, 4, 6, 8, 24, 48 and 72 h post dose. Plasma was separated by centrifugation of the blood at 4000 *g* for 5 min and transferred into clean glass test tubes. The samples were kept at −20 °C until assayed.

### Protein binding study

The unbound fraction of VCR in plasma was determined using ultrafiltration (Centrifree^®^, Amicon, Beverly, Massachusetts). Blood was obtained from NL, IHL and HL rats by exsanguination via cardiac puncture. For IHL and HL rats, blood was collected 72 and 36 h after i.p. doses of P407, respectively. The blood was centrifuged at 4000 *g* for 5 min. The NL, IHL and HL rat plasma were spiked with VCR methanolic solution to allow for final concentrations of 1 mg/L. The volume of methanol added to each tube did not exceed 0.05 % (v/v). Tubes were incubated for 1 h in a 37 °C water bath shaker. A volume of 1 mL of each tube was transferred to an ultrafiltration device (*n* = 4), (centrifree^®^, Millipore, Carrigtwohill, Ireland) which was then placed in a fixed angle centrifuge rotor and spun at 2000 *g* for 10 min at 37 °C. The samples were then analyzed for VCR concentration. In HL and IHL VCR protein binding test, the ultrafiltration devices were preincubated of with 5 % Triton^®^ (X-100) for 12 h to overcome the binding of drugs and lipids to the filter. The devices were then used after being rinsed several times with double-distilled water [11, 21].

### Assay

A validated HPLC assay was used for the measurement of VCR concentrations in plasma and Sorenson sodium phosphate buffer (pH 7.4) [21]. The validated lower limit of quantitation was 50 ng/mL based on 0.2 mL plasma or buffer. For standard curve construction, drug-free plasma and Sorenson sodium phosphate buffer (pH = 7.4) were used and spiked with appropriate amounts of VCR. The calibration curves relating peak area ratio to expected concentration were highly linear from 50 to 5000 ng/mL of VCR in NL, IHL, HL plasma and buffer (*r*^*2*^ > 0.997).

### Data and statistical analysis

Non-compartmental methods were applied to calculate the pharmacokinetic parameters. The elimination rate constant (λz) was calculated by subjecting the plasma concentrations in the terminal phase to linear regression analysis. The terminal phase t½ was calculated using the equation t½ =0.693/λz. The AUC_0-∞_ was calculated using the combined log-linear trapezoidal rule from time 0 h postdose to the time of the last measured concentration, plus the quotient of the last measured concentration divided by λz. The concentration at time 0 (C_0_) after i.v. dosing was estimated by back extrapolation of the log-linear regression line using the first three measured plasma concentrations to time. The clearance (Cl) was calculated as the quotient of dose to AUC_0-∞_ and the volume of distribution (Vβ) as the quotient of clearance by λz. Volume of distribution of the central compartment (Vc) is calculated as the quotient of dose by Co.

The plasma-unbound fraction was determined by dividing the VCR concentration in the filtrate by that measured in the prefiltered plasma. All compiled data were reported as mean ± SD, unless otherwise indicated. Significance of comparisons of means for the interactions of lipoprotein status and VCR PK, protein binding were assessed by one way analysis of variance (ANOVA), followed by Bonferroni post-hoc analysis (SPSS 22, SPSS Inc, Chicago, IL, USA). In all cases, the level of significance after Bonferroni adjustment was set at *p* = 0.016.

## Results and Discussion

**Fig. 1:**
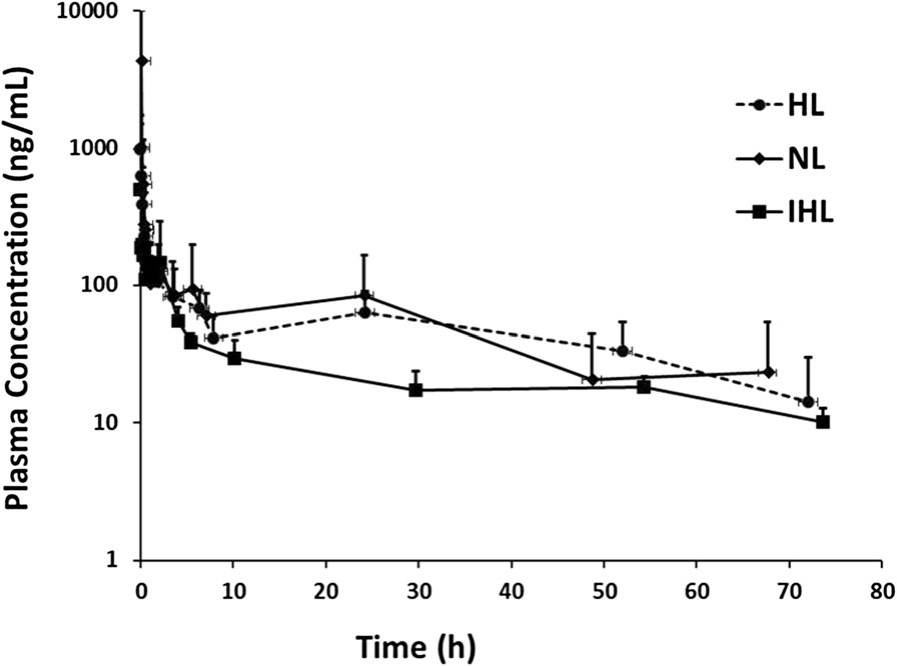
Comparative mean ± SD plasma concentration versus time curves of 0.15 mg/Kg intravenous vincristine sulphate (VCR) administrated in normolipidemic (NL), intermediate hyperlipidemic (IHL) or extreme hyperlipidemic (HL) rat groups

**Fig. 2:**
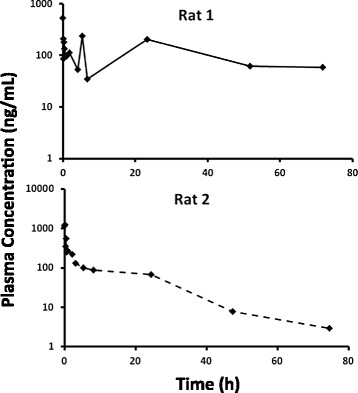
Plasma concentration versus time curves of 0.15 mg/Kg intravenous vincristine sulphate (VCR) administrated in normolipidemic (NL) rats showing two different decline patterns

After intravenous administration of 0.15 mg/kg of VCR to fasted rats, the plasma drug level decreased rapidly during the first 6–8 h, distribution phase, followed by a slower rate of decline till 72 h post i.v. with a mean elimination t½ of ~ 14 h (Fig. 1). VCR fast distribution phase followed by a slower elimination phase was had been previously described in the literature [1, 8]. A brief rise in concentration was observed at about 24 h which may indicate enterohepatic recirculation of the drug [22]. This was not uniform in all rats, where some rats showed this increase pattern while others demonstrated a regular concentration decline pattern (Fig. 2). This was reflected in the variability of the λz calculated (Table 1). Also it explains the variability in t½ and weight normalized Vβ (Table 1). It is worth mentioning that such variability in the decline in VCR plasma concentrations was consistent in all lipemic groups (NL, IHL and HL) which might be attributed to the inter-individual variability among rats. Inspite of this variability, the observed weight normalized Cl and Vc values were in complete agreement with those previously reported by ZHOU et al. (CL = 0.12 ± 0.08 L/Kg and Vc = 0.20 ± 0.11 L/h/Kg) (Table 1) [8].

**Table 1:** Pharmacokinetic parameters of vincristine sulphate after intravenous administration of 0.15 mg/kg solution in normolipidemic (NL), intermediate hyperlipidemic (IHL), and extreme hyperlipidemic (HL) rats

	NL	IHL	HL
Co (mg/L)	1.03 ± 0.50	0.50 ± 0.29	0.99 ± 0.76
AUC_0–∞_ (mg.h/L)	4.20 ± 3.23	2.25 ± 0.67	3.72 ± 1.38
λz (h^-1^)	0.06 ± 0.03	0.03 ± 0.01	0.04 ± 0.03
*t*_*½*_ (h)	14.6 ± 7.62	30.3 ± 11.6	28.2 ± 18.3
CL/kg (L/h/kg)	0.06 ± 0.04	0.07 ± 0.02	0.05 ± 0.02
V_β_/kg (L/kg)	1.53 ± 1.80	2.88 ± 0.52	1.74 ± 1.15
V_c_/kg (L/kg)	0.17 ± 0.08	0.39 ± 0.21	0.27 ± 0.22
fu	0.17 ± 0.04	0.19 ± 0.01	0.19 ± 0.07

To test for the effect of incremental increase in elevated plasma lipoproteins level on the VCR disposition, a P407-induced HL rat model was utilized. This HL induction method in rodents is an established method that is utilized by several research groups [12, 23–26]. It was first described by Johnston and coworkers [25, 27–29]. The P407 polymer, also known as pluronic F127, exerts its action through inhibiting plasma lipoprotein lipase and probably cholesterol 7α hydroxylase [27]. This model of HL is characterized by rapid onset, low toxicity, reversibility and ability to induce atherosclerosis in rodents with repeated doses [25]. It increased serum baseline corrected serum lipids in a nearly dose proportional fashion [30]. The maximum increase in lipids was observed at ~36 h, with most lipids remaining elevated for up to 120–180 h post i.p. dose [30, 31]. P407 was also recently proven to significantly increase serum leptin and decrease the serum adiponectin concentrations [30]. Most of the research laboratories utilizing this model start dosing their drugs 36 h post P407 ip injection and considering in their results that the increase in lipoprotein levels would be considerably higher than that clinically experienced by humans [14]. A recent publication studied the serum lipoproteins levels after a single 1 g/Kg ip dose of P407 and suggested the use of the model at time points later than 36 h when the lipoprotein concentrations will be more similar to those seen in hyperlipidemic patients [30]. As such, in our current study we have decided to study the effect of two levels of lipoprotein elevation (36 and 72 h post i.p. injection) on the pharmacokinetics and protein binding of VCR in rat.

VCR exhibits moderate binding affinity to plasma proteins [32], this was validated by the current protein binding results (~81–83 % bound). It showed a relatively small Vc (~0.17 L/Kg) and a larger Vβ (1.53 L/Kg) indicating good tissue distribution (Table 1).

The incremental increase in plasma lipoproteins levels resulted in no significant change in VCR total unbound fraction (fu, NL = IHL = HL) indicating the very low binding affinity of VCR to plasma lipoproteins (Table 1). This was also reflected by the similarity between C_0_ and Vc values among the different lipemic groups (Table 1). HL was reported to alter few metabolizing enzymes expression which made it necessary to comapre VCR AUC_0-∞_ and Cl values among rats with elevated lipoprotein level [11, 12, 16, 33]. Since VCR is an intermediate to low extraction ratio drug, it is expected that any slight change in the CYP P450 expression or VCR unbound fraction would result in a significant alteration of its hepatic clearance. However, in the current poloxamer-induced HL rat model, the incremental increase in plasma lipoproteins levels resulted in no significant change in VCR AUC_0-∞_ or weight normalized Cl among the different lipemic groups (Table 1 and Fig. 1). The results also show an increasing trend in t½ and weight normalized Vβ accompanying the increase in the lipid levels however, this was not statistically significant due to the variability in λz (Table 1).

Such results carries good information for ALL, malignant lymphoma and allogenic hematopoietic stem cell transplantation patients whom plasma lipoprotein levels were reported to increase during their therapy [19, 20]. It provides initial evidence that VCR does not have high binding affinity to plasma lipoproteins as such its unbound fraction, Co, as well as tissue distribution will not be highly affected by the transient elevation in the triglycerides levels reported. This would ensure less probability of VCR concentration based side effects, neurotoxicity, and better clinical efficiency due to proper tissue distribution [1, 3, 6]. VCR is eliminated mainly in bile and is metabolized through CYP 3A4 and 3A5 enzymes [1]. Drugs or diseases that affect those two elimination mechanisms through competitive or functional inhibition are expected to increase the VCR exposure in patients and increase its peripheral and/or central neurotoxic effect [3, 34]. It is worth mentioning, that VCR central neurotoxicity, seizures, results from SIADH leading to hyponatremia and currently researchers are investigating methods for prophylaxis and treatment of such toxicity [1, 4, 35, 36]. It is also important to mention that common genetic polymorphisms in CYP3A5 expression may contribute to the interpatient variability in VCR elimination [3]. In our HL induced rodent model we report no drug-disease interaction between elevated plasma lipoproteins and VCR elimination as such its exposure (AUC_0-∞_) and elimination phase t½ were not altered. However, it is important to note that the patient population we are targeting are usually exposed to complex drug regimens and exhibit modulated drug response. As such, despite HL by itself did not modulate VCR disposition, it could potentiate another drug-drug or drug-disease interaction involving VCR which warrants further study and monitoring.

## Conclusion

In conclusion, VCR demonstrated low affinity binding to plasma lipoproteins as such the incremental increase in plasma lipoprotein levels resulted in no significant alterations in VCR unbound fraction, initial plasma concentrations and volume of distribution. Induced HL also did not affect VCR elimination where similar VCR exposure (AUC_0-∞_) and elimination phase t½ were reported along the different lipemic groups. As such, ALL, malignant lymphoma and allogenic hematopoietic stem cell transplantation patients whom plasma triglycerides levels were reported to increase during therapy need not worry about HL-VCR interaction. Whether, HL can potentiate another drug-drug or drug-disease interaction involving VCR warrants further studying and monitoring to ensure therapeutic safety and efficiency in such immunocompromised population.

## References

[1] Said R, Tsimberidou AM (2014). Pharmacokinetic evaluation of vincristine for the treatment of lymphoid malignancies. Expert Opin Drug Metab Toxicol.

[2] Himes RH, Kersey RN, Heller-Bettinger I, Samson FE (1976). Action of the Vinca Alkaloids Vincristine, Vinblastine, and Desacetyl Vinblastine Amide on Microtubules in Vitro. Cancer Res.

[3] Hamdy DA, El-Geed H, El-Salem S, Zaidan M (2012). Posaconazole-vincristine coadministration triggers seizure in a young female adult: a case report. Case Rep Hematol.

[4] van Hasselt JG, van Eijkelenburg NK, Beijnen JH, Schellens JH, Huitema AD (2014). Design of a drug-drug interaction study of vincristine with azole antifungals in pediatric cancer patients using clinical trial simulation. Pediatr Blood Cancer.

[5] Martin J, Compston N (1963). Vincristine sulphate in the treatment of lymphoma and leukæmia. Lancet.

[6] Rosenthal S, Kaufman S (1974). Vincristine Neurotoxicity. Ann Intern Med.

[7] Dennison JB, Jones DR, Renbarger JL, Hall SD (2007). Effect of CYP3A5 expression on vincristine metabolism with human liver microsomes. J Pharmacol Exp Ther.

[8] Zhou XJ, Martin M, Placidi M, Cano JP, Rahmani R (1990). In vivo and in vitro pharmacokinetics and metabolism of vincaalkaloids in rat. II. Vinblastine and vincristine. Eur J Drug Metab Pharmacokinet.

[9] Wasan KM, Looije NA (2005). Emerging pharmacological approaches to the treatment of obesity. J Pharm Pharm Sci.

[10] Brown MS, Goldstein JL. Drugs used in the treatment of hyperliporoteinemias. Goodman and Gilman’s; The pharmacological basis of therapeutics, eighth ed. 1990; p. 874–896.

[11] Hamdy DA, Brocks DR (2011). Effect of hyperlipidemia on ketoconazole-midazolam drug-drug interaction in rat. J Pharm Sci.

[12] Hamdy DA, Brocks DR (2009). Experimental hyperlipidemia causes an increase in the electrocardiographic changes associated with amiodarone. J Cardiovasc Pharmacol.

[13] Mendis S, Puska P, Norrving B. Global atlas on cardiovascular disease prevention and control. Geneva : World Health Organization in collaboration with the World Heart Federation and the World Stroke Organization, c2011. for further details , proper reference is found at http://www.ncbi.nlm.nih.gov/nlmcatalog/101580439.

[14] Hamdy DA, Brocks DR (2011). The effect of increased lipoprotein levels on the pharmacokinetics of ketoconazole enantiomers in the rat. Xenobiotica.

[15] Shayeganpour A, Jun AS, Brocks DR (2005). Pharmacokinetics of Amiodarone in hyperlipidemic and simulated high fat-meal rat models. Biopharm Drug Dispos.

[16] Patel JP, Hamdy DA, El-kadi AO, Brocks DR (2012). Effect of serum lipoproteins on stereoselective halofantrine metabolism by rat hepatocytes. Chirality.

[17] Khalil HA, Elnaggar MM, Belal TS, El-Yazbi AF, Hamdy DA (2016). The effect of hyperlipidemia on the pharmacokinetics, hepatic and pulmonary uptake of posaconazole in rat. European Journal of Pharmaceutical Sciences..

[18] Bin Jardan YA, Brocks DR. The pharmacokinetics of dronedarone in normolipidemic and hyperlipidemic rats. Biopharm Drug Dispos. 2016. doi: 10.1002/bdd.2016. [Epub ahead of print] 10.1002/bdd.2016 27194397

[19] Blaser BW, Kim HT, Alyea EP, Ho VT, Cutler C, Armand P, Koreth J, Antin JH, Plutzky J, Soiffer RJ (2012). Hyperlipidemia and Statin Use after Allogeneic Hematopoietic Stem Cell Transplantation. Biol Blood Marrow Transplant.

[20] Tozuka M, Yamauchi K, Hidaka H, Nakabayashi T, Okumura N, Katsuyama T (1997). Characterization of hypertriglyceridemia induced by L-asparaginase therapy for acute lymphoblastic leukemia and malignant lymphoma. Ann Clin Lab Sci.

[21] Khalil HA, El-Yazbi AF, Belal TS, Hamdy DA (2015). High Performance Liquid Chromatographic Assay for the Simultaneous Determination of Posaconazole and Vincristine in Rat Plasma. Int J Anal Chem.

[22] Upmanyu R, Dvivedi J, Saxena Y (2009). Hepatotoxic effects of vincristine: an experimental study on albino rats. Indian J Physiol Pharmacol.

[23] Lee JH, Oh JH, Lee YJ (2011). Effects of experimental hyperlipidaemia on the pharmacokinetics of docetaxel in rats. Xenobiotica.

[24] Eliot LA, Jamali F (1999). Pharmacokinetics and pharmacodynamics of nifedipine in untreated and atorvastatin-treated hyperlipidemic rats. J Pharmacol Exp Ther.

[25] Palmer WK, Emeson EE, Johnston TP (1997). The poloxamer 407-induced hyperlipidemic atherogenic animal model. Med Sci Sports Exerc.

[26] Chhabria MT, Suhagia BN, Brahmkshatriya PS, Raval PM (2011). Synthesis and antihyperlipidemic activity of some novel 4-(substitutedamino)-5-substituted-3-mercapto- (4H)-1,2,4-triazoles. Arzneimittelforschung.

[27] Johnston TP, Palmer WK (1993). Mechanism of poloxamer 407-induced hypertriglyceridemia in the rat. Biochem Pharmacol.

[28] Wang YJ, Sun JB, Li F, Zhang SW (2006). Hyperlipidemia intensifies cerulein-induced acute pancreatitis associated with activation of protein kinase C in rats. World J Gastroenterol.

[29] Yousufzai SY, Siddiqi M (1976). 3-Hydroxy-3-methylglutaric acid and triton-induced hyperlipidemia in rats. Experientia.

[30] Chaudhary HR, Brocks DR (2013). The single dose poloxamer 407 model of hyperlipidemia; systemic effects on lipids assessed using pharmacokinetic methods, and its effects on adipokines. J Pharm Pharm Sci.

[31] Li C, Palmer WK, Johnston TP (1996). Disposition of poloxamer 407 in rats following a single intraperitoneal injection assessed using a simplified colorimetric assay. J Pharm Biomed Anal.

[32] Dennison JB. Vincristine Metabolism and the Role of Cyp3a5. 2007 10.1124/jpet.106.118471 17272675

[33] Patel JP, Brocks DR (2009). The effect of oral lipids and circulating lipoproteins on the metabolism of drugs. Expert Opin Drug Metab Toxicol.

[34] Moriyama B, Henning SA, Leung J, Falade-Nwulia O, Jarosinski P, Penzak SR, Walsh TJ (2012). Adverse interactions between antifungal azoles and vincristine: review and analysis of cases. Mycoses.

[35] Carella AM, Marinelli T, Di Pumpo MD, Ponziano E, Benvenuto A (2015). Metabolic Disorders in Hematologic Malignancies-A Review. Arch Med.

[36] Abouayana M, Naiel A, Hamdy DA. Possible protective role of erythropoietin in vincristine-induced central toxicity in rat. Clin Pharmacol Biopharm. 2015;4. http://www.omicsonline.org/proceedings/possible-protective-role-of-erythropoietin-in-vincristineinducedcentral-toxicity-in-rat-37510.html.

